# How to Produce an Alternative Carbon Source for Denitrification by Treating and Drastically Reducing Biological Sewage Sludge

**DOI:** 10.3390/membranes11120977

**Published:** 2021-12-12

**Authors:** Maria Cristina Collivignarelli, Alessandro Abbà, Francesca Maria Caccamo, Marco Carnevale Miino, Angela Durante, Stefano Bellazzi, Marco Baldi, Giorgio Bertanza

**Affiliations:** 1 Department of Civil Engineering and Architecture, University of Pavia, Via Ferrata 3, 27100 Pavia, Italy; francescamaria.caccamo01@universitadipavia.it (F.M.C.); marco.carnevalemiino01@universitadipavia.it (M.C.M.); stefano.bellazzi01@universitadipavia.it (S.B.); 2 Interdepartmental Centre for Water Research, University of Pavia, Via Ferrata 3, 27100 Pavia, Italy; 3 Department of Civil, Environmental, Architectural Engineering and Mathematics, University of Brescia, Via Branze 43, 25123 Brescia, Italy; alessandro.abba@unibs.it (A.A.); giorgio.bertanza@unibs.it (G.B.); 4 Freelance Chemist, Via Carducci 12, Casirate d’Adda, 24040 Bergamo, Italy; angela.durante@pec.chimici.it; 5 Department of Chemistry, University of Pavia, Viale Taramelli 12, 27100 Pavia, Italy; marco.baldi@unipv.it

**Keywords:** wastewater treatment plant, sludge minimization, carbon recovery, thermophilic membrane reactor, nitrate uptake rate tests, respirometric tests, circular economy

## Abstract

Minimizing the biological sewage sludge (BSS) produced by wastewater treatment plants (WWTPs) represents an increasingly difficult challenge. With this goal, tests on a semi-full scale Thermophilic Alternate Membrane Biological Reactor (ThAlMBR) were carried out for 12 months. ThAlMBR was applied both on thickened (TBSS) and digested biological sewage sludge (DBSS) with alternating aeration conditions, and emerged: (i) high COD removal yields (up to 90%), (ii) a low specific sludge production (0.02–0.05 kg_VS produced_/kg_CODremoved_), (iii) the possibility of recovery the aqueous carbon residue (permeate) in denitrification processes, replacing purchased external carbon sources. Based on the respirometric tests, an excellent biological treatability of the permeate by the mesophilic biomass was observed and the denitrification kinetics reached with the diluted permeate ((4.0 mgN-NO_3_^−^/(g_VSS_ h)) were found comparable to those of methanol (4.4 mgN-NO_3_^−^/(g_VSS_ h)). Moreover, thanks to the similar results obtained on TBSS and DBSS, ThAlMBR proved to be compatible with diverse sludge line points, ensuring in both cases an important sludge minimization.

## 1. Introduction

In the European Community, the gradual implementation of the urban wastewater (WW) directive determined an increasing quantity of biological sewage sludge (BSS) production due to a significant increase in discharges into public sewers and the request for greater efficiency of the purification process [1,2]. For the EU-27, in 2005 about 11 million tons of dry sludges were produced [3] and this amount is expected to have now exceeded 13 million tons of dry matter [4,5]. From the data published by ISPRA (Italian Superior Institute for Environmental Protection and Research) in 2018, the urban WW treatment activities produced approximately 3.1 million tons of sludge in Italy alone. Approximately 800 thousand tons generated by the treatment of industrial WW were also produced [6].

For Europe’s decisions oriented to circular economy processes, identification of long-term technical-economic strategies that are able to provide answers to this problem is very important [7]. First of all, it is necessary to follow the waste hierarchy detailed in Directive 2018/851/EC, which establishes the rules and priorities in the treatment and management of waste in order to ensure the lowest environmental impact: (i) prevention; (ii) preparation for use; (iii) recycling; (iv) energy recovery; and (v) final disposal [8]. As the legislation points out, the use of landfill disposal should be discouraged, as it is not an effective and efficient approach [9]. The reuse of BSS in soils and the incineration appear to be the main routes adopted [4].

In Europe, in 2015, despite the strong regulatory uncertainties, the main destination was represented by spreading in agriculture (45%), followed by incineration (27%), composting and other forms of reuse (13%), disposal in landfills (8%), and other forms of disposal (3%) [3]. In 2016, in Italy, according to the data published by ARERA (Regulatory Authority for Energy, Networks and the Environment), the BSS recovery has exceeded the disposal (almost, 80% vs. 20%). The 70% of recovered BSS were reused in agriculture, by direct spreading or by composting amending products, while a residual percentage was destined for co-incineration in waste-to-energy plants or cement factories [10].

The prevailing operation of reuse BSS in agriculture is not exempt from critical points such as the presence of harmful substances originally contained in WW [11,12,13] and the low acceptance by the population, mainly related to the odor impact [14,15]. Furthermore, for instance in Italy, several managers of wastewater treatment plants (WWTPs) reported increasing difficulties in the reuse of BSS as soils conditioner due to the absence of adequate outlets in their respective territories. This has led to a resumption of landfilling or an increase in extra-regional and cross-border flows [10].

Considering that BSS disposal represents a deep problem in the environmental sector, technologies that minimize BSS are of fundamental importance [16]. BSS minimization can be achieved through two main approaches: (i) reducing the production in water line of WWTPs, or (ii) applying technologies in sludge line acting on BSS already produced by biological processes [17].

In this study, results of an innovative biological process applied in sludge line are presented. The Thermophilic Alternate Membrane Biological Reactor (ThAlMBR) is an advanced biological membrane system used to lysate and oxidize the excess BSS produced by WWTP through thermophilic bacteria, under controlled conditions of temperature and aeration. Previous experiments on ThAlMBR pilot plant mainly concerned the treatment of industrial aqueous waste [18,19,20]. With regard to BSS, only two preliminary experiments have been carried out on TBSS [21,22], never on DBSS. A recent experimentation was conducted on TBSS to evaluate the minimization of BSS produced by a municipal WWTP owing to the application of ThAlMBR. The reduction of BSS was quantified in 89% and 92%, in terms of total and volatile solids fed to the system, respectively [23]. These previous studies have made it possible to approach the optimal operative conditions for the process (hydraulic retention time, organic loading rate (OLR), temperature, etc.), which, however, have never been thoroughly tested for long periods, as was the case in this experimentation which lasted 12 months.

Furthermore, there are no full scale plants equipped with this technology for the treatment of BSS while two aqueous waste treatment plants have been built. The first plant was able to remove almost 90% of COD with a specific sludge production of 0.08–0.09 kg_VSSproduced_/kg_CODremoved_ (VSS: volatile suspended solids) [24]. The second full scale removed 78.2% of COD (operating with an organic loading rate (OLR) between 1.5 and 2 kg/(m^3^d), and almost 82% of COD in case of an OLR greater than 3 kg/(m^3^d). In this case, a specific sludge production of 0.052 kg_VSproduced_/kg_CODremoved_ was highlighted [25].

Regardless of the substrate being fed, ThAlMBR produces a carbonaceous aqueous residue (permeate). In recent work, the feasibility of reuse permeate, from ThAlMBR fed with aqueous waste, in denitrification processes as alternative carbon source was proved [25]. If the amount of organic substance in the untreated WW is too low in relation to the required COD:N ratio, the addition of an external carbon source becomes necessary to increase the yields of the denitrification process [26,27]. This criticality can occur with post-denitrification but also with pre-denitrification schemes where the incoming effluent is particularly lacking in organic matter. Generally, the purchase of an external carbon source (e.g., methanol, ethanol, acetic acid, glucose), necessary to increase denitrification kinetics [27,28] represents the most significant costs in WWTPs management together with the disposal of the BSS produced [29,30]. 

As alternative sources of carbon, in order to guarantee economic savings and a lower environmental impact, WW from food, confectionery, dairy, and beverage production processes can be used, owing to the high content of organic carbon that is easily biodegradable [31,32]. An important disadvantage could be the inconstancy of both qualitative and quantitative characteristics of this industrial WW, related to the variety of production cycles [28].

In this work, biological treatability, with oxygen uptake rate (OUR) tests, and denitrification kinetics, with nitrate uptake rate (NUR) tests, were evaluated. The feasibility of reusing the permeate within the WWTP itself as an alternative caron source in denitrification, to move forward a water resource recovery facility (WRRF), from the limited vision of a WWTP intended for WW treatment only [33,34] was explored.

Moreover, the minimization of BSS production by means of ThAlMBR was investigated first, focusing also on its possible placement in the WWTP sludge line. Subsequently, attention was paid to the possible total or partial replacement of external carbon sources in the WWTP water line. Tests on a semi-full scale plant were carried out for 12 months. ThAlMBR was applied both on thickened (TBSS) and digested biological sewage sludge (DBSS).

## 2. Materials and Methods

### 2.1. ThAlMBR Pilot Plant

ThAlMBR pilot plant consisted of a thermally insulated biological reactor (volume: 1 m^3^). An ultrafiltration (UF) system allowed the separation of the oxidized aqueous residue (permeate) from the active thermophilic biomass. The UF retentate could be either recirculated upstream of the UF membranes or introduced into the thermophilic biological reactor. The membrane line included seven tubular ceramic membranes with a molecular cut-off of 300 kDa. More detailed information was reported by Collivignarelli et al. [19]. The presence of materials with a particle size of the order of a few mm could obstruct the membranes, therefore, before entering the UF unit and in correspondence with the recirculation line, bag filters are positioned to ensure a coarse filtration. 

The experimentation was conducted alternating aerobic/anoxic conditions to promote cell lysis processes and biological oxidation of the fed BSS. Due to the formation of biological foams, aerobic/anoxic cycles were balanced preliminary testing diverse solutions. 2 h of anoxic conditions followed by 6 h of aerobic conditions represent the optimal and stable solution in terms of reduction of biological foams and was chosen to perform the tests. During the aerobic phases, ThAlMBR worked with pure oxygen injected in the recirculation line. The dissolved oxygen and the temperature of the biological reactor were monitored by a submerged probe (Oxymax W COS31, Endress + Hauser, Reinach, Switzerland), and maintained between 2–6 mg/L and 47–53 °C for oxygen and temperature, respectively.

The system worked in autothermal mode owing to the development of heat produced by the oxidation reactions of the BOD (exothermic reactions). The plant was also equipped with a heat exchanger, necessary for cooling the reactor in case the exothermic oxidation reactions raise the temperature over 50 °C.

This work considers results obtained only during stable operating conditions, neglecting the fluctuations due to the start-up phase. 

### 2.2. ThAlMBR Placement in WWTP Sludge Line

**Figure 1:**
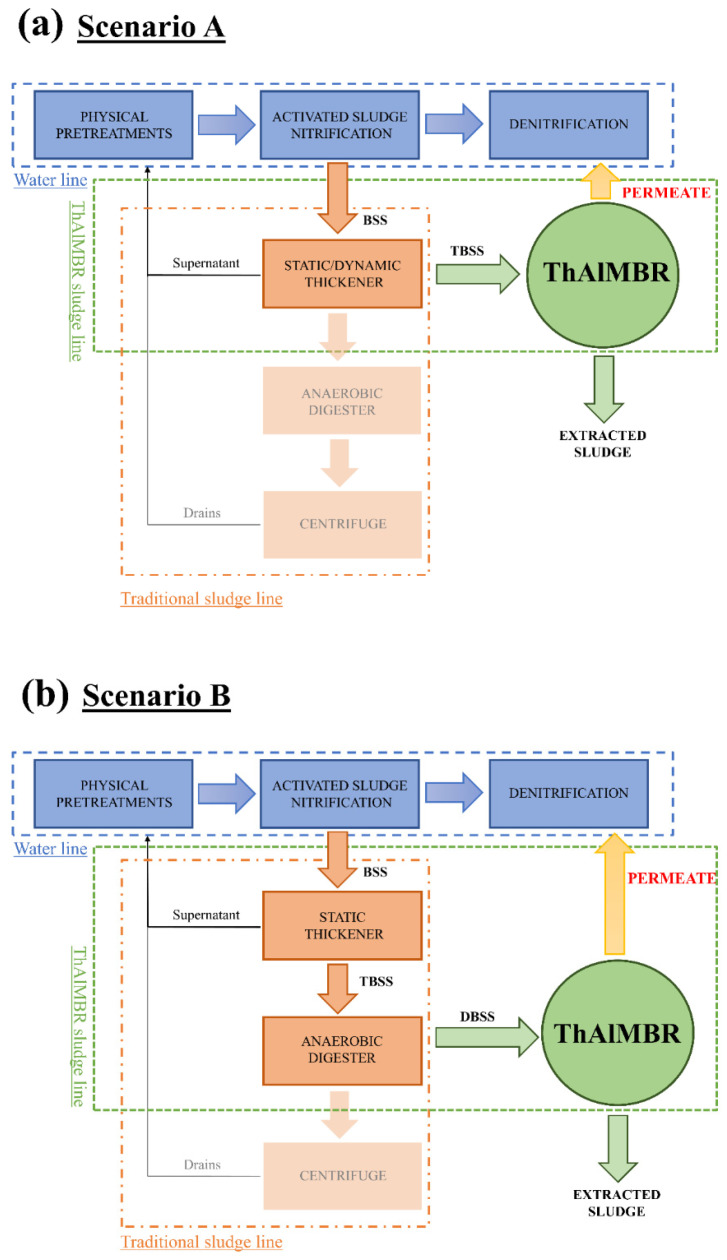
Applications of ThAlMBR in sludge line. (**a**) Scenario A: downstream of the thickener, (**b**) Scenario B: downstream of the anaerobic digester. BSS: biological sewage sludge, TBSS: thickened biological sewage sludge, DBSS: digested biological sewage sludge, VS: volatile solids.

ThAlMBR pilot plant was tested in two urban WWTPs located in Northern Italy (130,000 and 70,000 population equivalent, respectively). Two WWTPs with conventional active sludge (CAS) system, with a complete sludge line, and without industrial drains in WW entering the WWTPs, were chosen. As shown in Figure 1, two scenarios were identified as the ThAlMBR was introduced at diverse points of sludge lines to evaluate the feasibility of the process in minimizing the BSS. In scenario A (Figure 1a), the sludge line featured a static thickener, a dynamic thickener with polyelectrolyte dosage, an anaerobic digester, and a dewatering treatment by centrifuge. In this scenario, the pilot plant was placed downstream of the static thickener. In Scenario B (Figure 1b), ThAlMBR was placed downstream of the anaerobic digester.

In both cases, the permeate was investigated as alternative carbon source for denitrification processes.

### 2.3. TBSS and DBSS Treated by ThAlMBR

TBSS was sampled after the static thickener of plant A, before the addition of polyelectrolyte as a flocculant at the dynamic thickener (Scenario A, Figure 1a). DBSS was sampled after the anaerobic digestion of BSS produced by plant B (Scenario B, Figure 1b).

Table 1 shows the chemical and chemical-physical parameters of the two BSS fed to the process. The average values of the measurements taken during the experimentation, and the confidence interval were reported.

These substrates were almost equivalent from a chemical point of view. The only difference concerned the value of the inert solid residue, greater in DBSS due to the accumulation of inert material in the digester where samples were taken, and the ammoniacal nitrogen concentration, more than double in TBSS. The pH of TBSS was slightly more acidic, but this had no effect on the pH of the process which has settled around neutrality (Table 2). 

### 2.4. Operative Conditions

Table 2 shows the operative conditions inside ThAlMBR during the experimentation with TBSS and DBSS.

TBSS and DBSS were fed continuously for 10 months and 2 months, respectively. Initially, only the minimization of TBSS and the reuse of the permeate produced were taken into consideration. Following the obtained results, it was decided to deepen the study on the treatment of DBSS to evaluate the possible double applicability of ThAlMBR, representing the second phase a consolidation of an already tested process on TBSS. This explains the diverse duration of the two experimentation phases.

The total solids (TS) in the reactor were kept no more than 80 kg/m^3^ and their increase was managed through controlled extractions of thermophilic sludge. In fact, a total solids value greater than 100 kg/m^3^ could have compromised the hydrodynamics, the mechanicals, and the hydraulics of the pilot plant, as well as the change in rheological characteristics of the thermophilic sludge with consequent management problems.

In both sludges the OLR was the same (almost 3 kg_COD_/(m^3^d)). The hydraulic retention time (HRT) of the system was maintained around 10 d. The pH was close to neutrality, an optimum condition for thermophilic bacterial species.

### 2.5. Analytical Methods

The analytical parameters, in the fed TBSS and DBSS, in the thermophilic sludge and in the permeate, were monitored using official methods recognized internationally. COD was determined with the method proposed by ISO 6060: 1989 [35], total nitrogen (TN) was monitored with CNR-IRSA [36]. For ammoniacal nitrogen (N-NH_4_^+^) the method of APAT IRSA-CNR 4030 A1:2003 was used [37]. Organic nitrogen was calculated starting from the measurements of TN and N-NH_4_^+^, the nitric and nitrous nitrogen being negligible. Total solids (TS) were determined using UNI EN 14346:2007 [38] and volatile solids using UNI EN 15169:2007 [39]. 

Electrical conductivity was daily measured using the probe WTW-IDS, model TetraCon^®^ 925 (Xylem Analytics Germany Sales GmbH & Co, Mainz, Germany). pH was daily measured using the probe WTW-IDS, Model SenTix^®^ 940 (Xylem Analytics Germany Sales GmbH & Co, Mainz, Germany).

### 2.6. Respirometric Tests

#### 2.6.1. OUR Tests

OUR tests were performed as an index of metabolic-enzymatic activity of a biological system, as suggested by Hagman et al. [40] and Kristensen et al. [41]. The respirometric tests of OUR were used to evaluate the biological treatability of the permeate in the mesophilic field. A mesophilic biomass from a traditional CAS process was used and endogenous OUR tests with biomass alone were first conducted to understand its health status. Subsequently, exogenous OUR tests were carried out by contacting the mesophilic biomass and the substrates (BSS fed or ThAlMBR permeate), in equal volume ratio. Total of 500 mL of biomass was aerated for 30 min up to a dissolved oxygen concentration of about 7 mg/L and then the aeration was stopped, mixed with 500 mL of substrate and the beaker was hermetically closed to prevent the entry of oxygen from the external environment. The substrate either undiluted or suitably diluted with distilled water was added with the aim of evaluating a possible toxic-inhibiting effect of the substrate against the mesophilic biomass. Continuous stirring was maintained (about 400 RPM). All tests took place at room temperature. The OUR value was calculated considering the concentration of VSS in the tested sample and the slope of the oxygen consumption curve [41].

To globally assess the performance of ThAlMBR process, an index has been proposed, GPI (global process index), which is a dimensionless number between −1 and 2. In particular, the index aims to investigate the increase/decrease in the permeate biodegradability compared to the BSS fed to ThAlMBR, in relation to the COD removal performance in ThAlMBR process. The Equation (1) has been used to calculate the GPI:

(1) $$\mathrm{GPI}\;\left[-\right]=\frac{{\mathrm{OUR}}_{\mathrm{OUT}}\;-{\;\mathrm{OUR}}_{\mathrm{IN}}}{{\mathrm{OUR}}_{\mathrm{OUT}}+{\mathrm{OUR}}_{\mathrm{IN}}}+\frac{{\mathrm{COD}}_{\mathrm{IN}}\;-{\;\mathrm{COD}}_{\mathrm{OUT}}}{{\mathrm{COD}}_{\mathrm{IN}}+{\;\mathrm{COD}}_{\mathrm{OUT}}}$$

where:

- OUR_IN_ [mg_O_2__/(g_VSS_ h)] represents the OUR of the BSS fed to ThAlMBR;
- OUR_OUT_ [mg_O_2__/(g_VSS_ h)] represents the OUR of ThAlMBR permeate;
- COD_IN_ [mg/L] represents the COD of the BSS fed to ThAlMBR;
- COD_OUT_ [mg/L] represents the COD of ThAlMBR permeate.

The more the GPI value was close to 2, the greater was the biodegradability of the permeate compared to that of the starting BSS and the removal of COD carried out by ThAlMBR in the BSS. The more the index value was close to −1 the more the carbon in the permeate showed a poor biological degradation (certainly lower than that of BSS fed to ThAlMBR). GPI remained almost 1 if there is: (i) an excellent degradation of COD with a reduction in the biodegradability of the permeate, or on the contrary, (ii) a reduced reduction of organic carbon and an important improvement of the biodegradation of the permeate. The latter case can be linked to the presence of organic substance which is difficult to biodegrade by the thermophilic biomass in the BSS fed to the process. However, during the stay of the BSS in the reactor, the thermophilic biomass should be able to simplify the structure of the COD making it easily biodegradable in the permeate.

The reference substrate was represented by the BSS entering ThAlMBR. Therefore, with the GPI index it is possible to evaluate how ThAlMBR modified the biodegradability of the treated BSS. 

#### 2.6.2. NUR Tests

NUR tests were performed to evaluate the feasibility of using the permeate as an external source of organic carbon for denitrifying bacteria. The biomass used was taken from a denitrification process present in a real WWTP and both methanol (as an external source of organic carbon of reference) and the permeate suitably diluted with distilled water were used as substrates. Ammonia in permeate was stripped before tests to avoid possible inhibition of microorganism. The method described also in our previous study was used [25]. Total of 500 mL of biomass was aerated for 30 min and mixed with 500 mL of substrate, enriched with nitrates using KNO_3_ to obtain an initial N-NO_x_ concentration in the starting sample of approximately 50 mg/L. The system was kept in constant stirring (about 400 RPM) and was hermetically sealed to avoid the solubilization of atmospheric oxygen. The pH was maintained around neutrality with the addition of H_2_SO_4_ if necessary. The tests lasted a total of 6 h, and every hour 25 mL of sample was taken and filtered for chemical analyses (COD, N-NO_x_^−^). The NUR was evaluated considering the concentration of VSS in the tested sample and the slope of the nitric and nitrous nitrogen consumption curve was determined.

## 3. Results and Discussion

### 3.1. Performance and Sludge Minimization

**Figure 2:**
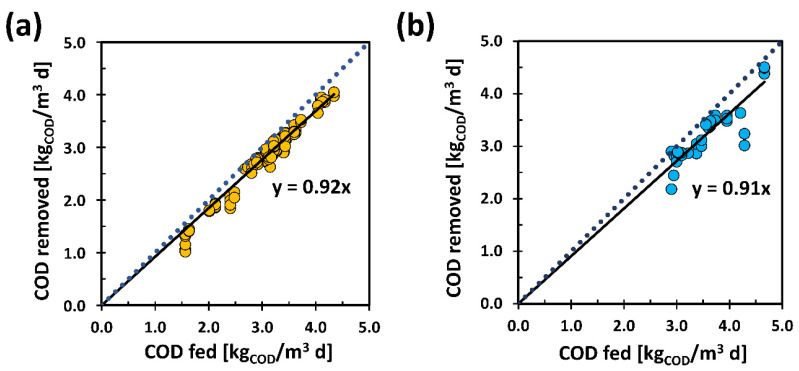
COD removal yields with TBSS (**a**) and DBSS (**b**). The continuous black lines represent the fitting of COD removed as a function of COD fed while the dot blue lines represent the condition of complete COD removal.

The organic substrate fed was oxidized by the thermophilic bacteria present inside the reactor. Figure 2 shows the COD removal yields calculated comparing the inlet (TBSS and DBSS) and outlet (permeate) concentrations from the process. Specifically, Figure 2a shows the COD removal in the first part of the experiment with TBSS, in which an average removal efficiency of 92% was achieved. While Figure 2b shows the COD removal yields in DBSS, with an average pilot plant performance of 91%. The results relating to the COD removal yields are comparable between the two sludges. As there were no particularly noticeable differences, it is possible to state the excellent applicability of the ThAlMBR to both TBSS and DBSS.

It is important to underline that the COD of the permeate did not have significant variations in terms of concentration (2318 ± 155 mg_COD_/L and 3400 ± 855 mg_COD_/L, in case of TBSS and DBSS, respectively). This result was indicative of a stable operation of the process which ensured an output permeate with uniform and unchanged qualitative and quantitative characteristics. It constituted a not negligible aspect in view of a possible reuse of the aqueous residue which continued to maintain an important residual COD considering the treatment with a mesophilic biomass.

The results reported in Figure 2 were obtained under comparable conditions of average OLR: 3.2 ± 1.7 kg_COD_IN__/(m^3^ d) for TBSS and 3.4 ± 0.2 kg_COD_IN__/(m^3^ d) for DBSS. The COD removal yields obtained with ThAlMBR were completely similar or superior to those of the MBR systems reported in the scientific literature. For example, a COD removal efficiency of 62–79% was found treating landfill leachate with an aerobic thermophilic MBR [42]. From other types of aqueous waste, ThAlMBR removed almost 78% of COD with an OLR of 3–6 kg_COD_IN__/(m^3^ d) [43] and proved to be able to remove up to 94% with HRT of 10 d [20]. Considering the treatment of thickened biological sludge by means of a thermophilic process with alternating oxygen cycles, COD removal values of 57% were achieved with an average HRT of 20 days and an OLR of approximately 1.4–1.8 kg_COD_IN__/(m^3^ d) [21]; even higher up to 85% with HRT of 13–14 days and OLR of almost 2 kg_COD_IN__/(m^3^ d) [22].

**Figure 3:**
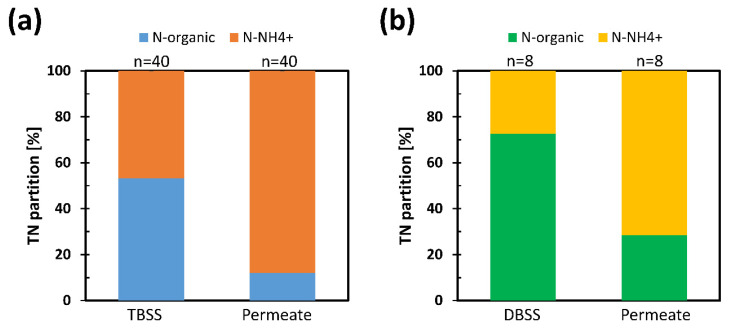
TN partition (**a**) in TBSS and (**b**) in DBSS. TN: total nitrogen, TBSS: thickened biological sewage sludge, DBSS: digested biological sewage sludge, n: number of data.

ThAlMBR is usually a powerful ammonia producer, as has already been observed in previous experiments [18,22], capable of converting organic nitrogen into ammonia through transamination reactions [44]. Similar behavior was also observed in this experiment on TBSS and DBSS. The conversion of organic nitrogen, introduced with BSS, into ammoniacal nitrogen is clearly visible in Figure 3. The subdivision of total nitrogen into its various forms has been represented, as average values on the experimental phases. The contribution of N-NO_X_ during the experimentation had a negligible weight given its concentrations involved between 1 and 10 mg/L. Therefore, in the distribution of nitrogen forms, only the forms of organic and ammoniacal nitrogen were considered.

**Figure 4:**
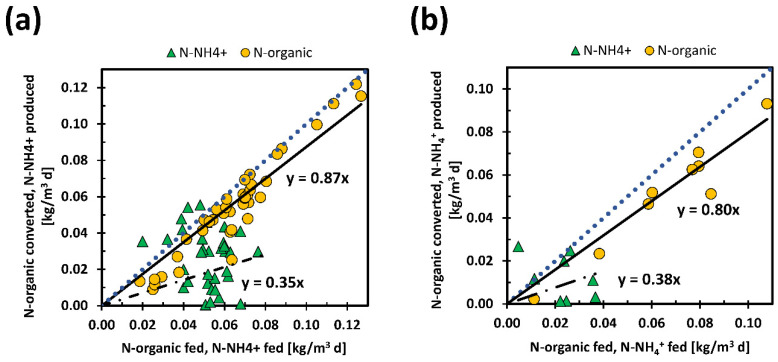
Conversion rates of N-organic in N-NH_4_^+^ with TBSS (**a**) and with DBSS (**b**). The black lines represent the fittings while the dot blue lines represent the condition of complete pollutant removal.

In TBSS, the total nitrogen entering ThAlMBR is made up equally of organic and ammoniacal nitrogen, while in DBSS the prevalent form of nitrogen is organic (about 70%). In both cases, the ammonification phenomena due to the thermophilic biomass is evident in the permeate output. The ammonification process was investigated in more detail by evaluating the production yields of ammoniacal nitrogen and calculating the amount of organic nitrogen converted. The results are shown in Figure 4.

A reduction of organic nitrogen between inlet and outlet in the 80–90% range was evaluated, 87% and 80% for TBSS and DBSS, respectively. About ammoniacal nitrogen, production yields of 35% for TBSS and 38% for DBSS were obtained. In ThAlMBR, the amount of ammonia produced should not deviate much from the amount of organic nitrogen converted, although percentage differences of approximately 40–50% were observed in this experimentation. A portion of the organic nitrogen was certainly used by thermophilic bacteria for metabolic functions, but, in particular the production of ammoniacal nitrogen seemed to be underestimated, as was also observed in our previous tests [45]. This can be attributed to the stripping phenomena to which ammonia was subjected following the high temperatures and pH of the process, sometimes higher than neutrality.

For the evaluation of the specific sludge production in ThAlMBR, no distinction between the periods of feeding TBSS and DBSS has been done, as the process did not present significant differences. For the calculation of the specific production of sludge (i) the thermophilic biomass extractions carried out to keep the value of TS of the process approximately constant and (ii) the quantity of sludge lost during ordinary and extraordinary maintenance operations of the plant were considered. Ordinary maintenance operations mainly concerned the daily cleaning of the pre-filters present both in the recirculation line and in the UF line. The specific sludge production was therefore calculated considering, in addition to the TS and vs. extracted and lost, also the COD removed. ThAlMBR sludge production was 0.05–0.08 kg_TSproduced_/kg_CODremoved_ and 0.02–0.05 kg_VSproduced_/kg_CODremoved_. Results were comparable to those of some previous experiments on industrial aqueous waste: 0.04 kg_VSproduced_/kg_CODremoved_ [19,45] and 0.09 kg_VSproduced_/kg_CODremoved_ [18]. However, the results of this experimentation were better than those reported by Simstich et al. [46] for thermophilic aerobic MBRs where the specific sludge production were 0.07–0.29 kg_MLSS_/kg_CODremoved_ and 0.03–0.09 kg_MLVSS_/kg_CODremoved_ (MLSS: mixed liquor suspended solids, MLVSS: mixed liquor volatile suspended solids).

For this same type of process, Suvilampi and Rintala [47] indicated values equal to 0.12–0.16 kg_TSS_/kg_CODremoved_. On the other hand, for a mesophilic MBR the sludge production can be generally higher than the thermophilic conditions, such as 0.10 kg_VSSproduced_/kg_CODremoved_ [48] up to 0.19 kg_VSSproduced_/kg_CODremoved_ [49].

### 3.2. OUR Tests 

**Figure 5:**
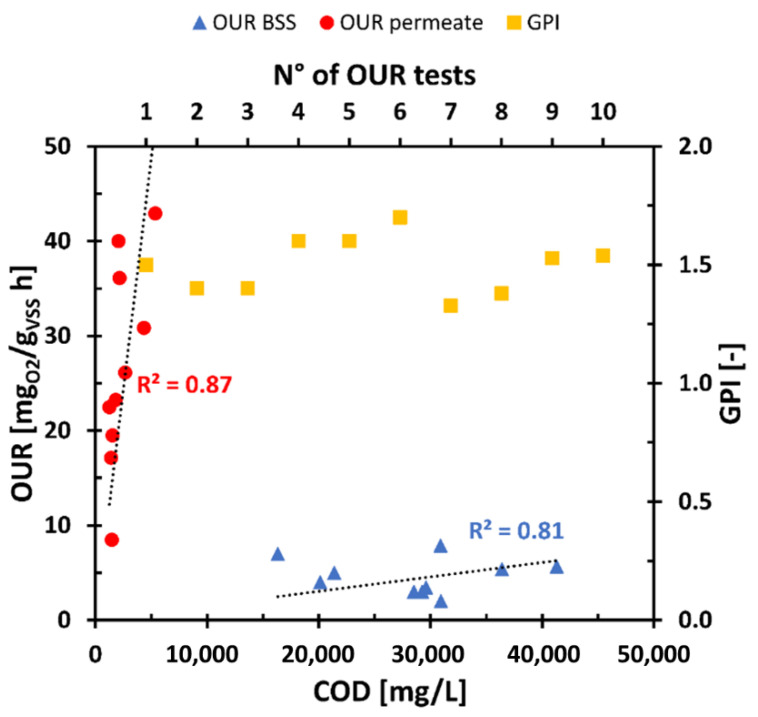
Trend of OUR and GPI. Dot black lines represent the linear fitting of OUR values as a function of COD. Yellow squares indicate the GPI values in ten diverse OUR tests.

Aerobic biomass uses oxygen to perform catabolic/anabolic functions and to activate the biological oxidation processes of organic pollutants. For comparing the biodegradability of different substrates, OUR data are an important tool. Highly biodegradable substrates determined a high demand for oxygen in the short term, on the other hand, poorly biodegradable substrates have much lower oxygen consumption rates. One of the objectives of the experiment was to evaluate the possible reuse of the permeate as an external carbon source in a denitrification process in a WWTP water line. Therefore, the biodegradability of the aqueous residue produced has been compared with that of the fed substrate. OUR tests were performed using a mesophilic biomass taken from a CAS system. In the discussion of the results, no distinction is made between TBSS and DBSS as the trend of the results was comparable. Figure 5 shows the results of the OUR and GPI values obtained from the various tests.

The results obtained showed that OUR of BSS fed was always lower than the output permeate. OUR of the permeate reached an average value of 31.1 ± 6.6 mg_O_2__/(g_VSS_ h) against an average value of 4.0 ± 1.1 mg_O_2__/(g_VSS_ h) of the fed BSS. Similar results were obtained by Collivignarelli et al. [18], using a permeate deriving from the treatment of industrial aqueous waste. This difference between inlet and outlet to the reactor seemed to be due to an increase in biodegradability achieved by the thermophilic process. Despite the important reduction of COD carried out by ThAlMBR, the permeate contained residual organic substances highly biodegradable from the mesophilic biomass. ThAlMBR oxidize the fraction of COD more difficultly biodegradable from a mesophilic biomass, leaving in the permeate substances more easily biodegradable from a CAS.

The GPI values were also shown in Figure 5. GPI has always assumed positive values, between 1.25 and 1.5. These results showed a constant trend of the excellent performance of the process, both in terms of COD removal and increase in biodegradability. In this way, the excellent availability of usable organic carbon from a mesophilic biomass in the permeate was confirmed, already visible from the OUR values.

The most important result was the demonstration of an increase in the biodegradability of the permeate compared to that of the fed BSS. The COD of the permeate (around 2000–3000 mg/L), despite being of an order of magnitude lower than the COD of the fed BSS, was found to be more degradable by the mesophilic biomass. Therefore, ThAlMBR guaranteed an important reduction of COD but at the same time remaining organic substance was highly biodegradable promoting possible reuse of the permeate.

**Figure 6:**
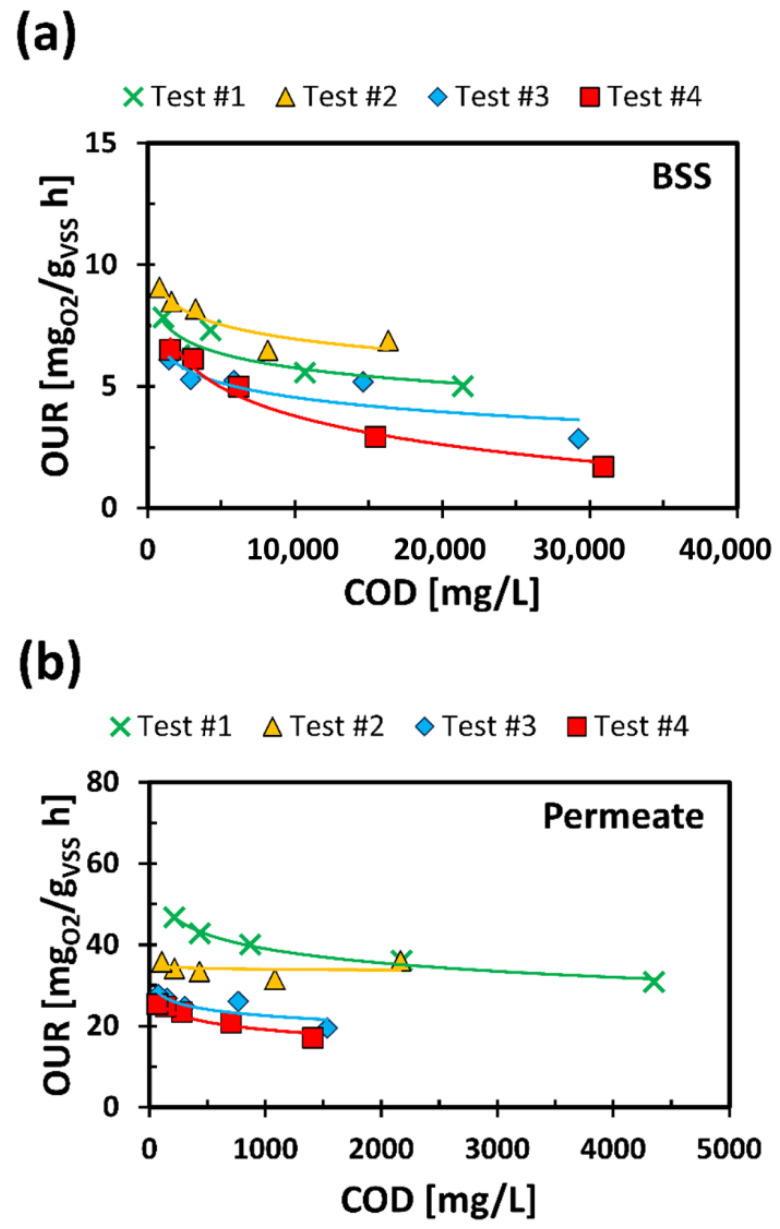
OUR trend with COD, for fed BSS (**a**) and permeate (**b**). Continuous lines represent the fitting of OR as a function of COD.

Any inhibiting substances present within the permeate, resulting from the catabolism processes of COD, can affect the OUR value. In this regard, respirometric tests were conducted on the fed BSS and the permeate diluted at diverse concentrations. The trends are shown in Figure 6.

Each curve represents a test carried out on the same BSS or permeate sample, respectively. For each test, the sample was tested not diluted (associated with the major COD), and at least with three dilutions (COD decreasing with the dilution increasing). As the dilution factor increased, the OUR values increased and therefore the biodegradability of the tested substrates. Trends of this type are characteristic of substrates with a toxic-inhibiting effect on biomass [50]. This effect is linked to the presence of harmful substances in the tested substrate which could disturb and, in the worst cases, inhibit the biomass put in contact with the substrate. The results showed that the toxic-inhibiting effect can be reduced by suitably diluting the analyzed substrate and the increase in OUR with the dilutions was not excessively marked, demonstrating a reduced acute toxicity for mesophilic biomass. 

The feedback obtained from OUR tests, in addition to supporting the thesis of a good complementarity between CAS system and a thermophilic process, has shown that ThAlMBR process does not worsen the toxic-inhibiting effect of the permeate against a traditional mesophilic biomass, compared to those of BSS.

### 3.3. NUR Tests

**Figure 7:**
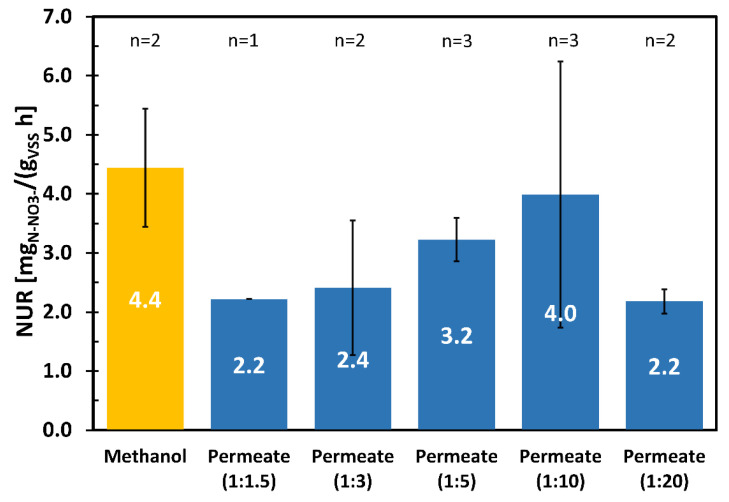
Results of NUR tests. Each test was conducted with the substrate fed to denitrification. The dilution value has been indicated in brackets. n: number of tests.

Through the NUR tests it was possible to verify the effect of the permeate on the denitrifying biomass, which is an important aspect in case of reusing the permeate as an external source of carbon in denitrification process. As for the OUR, NUR tests were performed by diluting the permeate. For the dilutions, the water entering the denitrification section of a full scale WWTP was used to recreate the real operating conditions. The results of the NURs are reported in Figure 7.

The substrates used in the NUR tests were permeate in diverse dilutions and methanol. The latter was a highly biodegradable carbonaceous substrate used as a reference, usually purchased in the WWTPs to increase the denitrification kinetics in the post-denitrification processes. The test carried out with methanol could represent a typical situation found in a real WWTP. Different permeate dilutions were tested: 1:1.5, 1:3, 1:5, 1:10, 1:20.

The highest NUR value was obtained with methanol. Of all tests carried out with the permeate, the best result was obtained by diluting the permeate 1:10, as it is comparable with the NUR value of methanol. Tests carried out at lower permeate dilutions (1:1.5; 1:3; 1:5) showed a lower denitrification rate, probably due to a slight toxic-inhibiting effect of the substrate on mesophilic bacteria (resolved by increasing the dilution). The excessive dilution of the permeate (1:20) determined a lower NUR probably due to a low amount of bioavailable COD, necessary for heterotrophic bacteria to carry out denitrification. 

The results obtained proved that the permeate can be potentially used as an alternative carbon source in a denitrification process, with results not significantly different from those obtained using methanol. Furthermore, the NUR of the permeate was found to be in line with and in some cases higher than those found in the literature using other types of industrial WW (Table S1).

## 4. Conclusions

The results of the semi-full scale ThAlMBR allowed to confirm its applicability in the minimization of BSS. In addition, the versatility of the technology was demonstrated for the first time by testing a double location in WWTP sludge lines, both downstream of a thickener and downstream of an anaerobic digester. Specific production of sludge between 0.02 and 0.05 kg_VSproduced_/kg_CODremoved_ was obtained. The possible recovery of the permeate aqueous residue deriving from ThAlMBR was then investigated by means of OUR and NUR tests. The permeate, owing to (i) its constant qualitative and quantitative characteristics, (ii) its excellent biological treatability in the mesophilic field, and (iii) denitrification kinetics comparable to those obtained using methanol (4.0 mgN-NO_3_^−^/(g_VSS_ h) with a dilution ratio of 1:10), can be advantageously reused as an alternative carbon source in a post-denitrification process. After stripping in an acid tower for ammonia recovery, the permeate could be recirculated in the WWTP water line hosting ThAlMBR. In this way (i) economic savings, (ii) greater storage safety, and (iii) recovery operation in a circular economy approach applied to water treatment can be guaranteed.

## Figures and Tables

**Table 1:** Chemical and physico-chemical parameters of TBSS and DBSS.

Parameter	TBSS	DBSS
	Mean Value ± Confidence	Mean Value ± Confidence
COD [mg/L]	32,348 ± 1488	35,223 ± 2706
TN [mg/L]	1173 ± 84	914 ± 260
N-NH_4_^+^ [mg/L]	548 ± 51	251 ± 77
N-NO_x_ [mg/L]	3.4 ± 1.2	n.d.
TS [g/L]	20 ± 2	24 ± 4
VS [g/L]	14 ± 1	14 ± 2
VS/TS [%]	71 ± 2	57 ± 4
pH [-]	5.5 ± 0.1	6.5 ± 0.3
Electrical conductivity [µS/cm]	2586 ± 182	3345 ± 439

COD: chemical oxygen demand; TN: total nitrogen; TS: total solids; VS: volatile solids; n.d.: not detected.

**Table 2:** Operative conditions in the thermophilic reactor with TBSS and DBSS.

Operative Parameter	TBSS	DBSS
	Mean Value ± Confidence	Mean Value ± Confidence
TS [kg/m^3^]	62 ± 2	77 ± 6
VS [kg/m^3^]	28 ± 1	30 ± 2
VS/TS [%]	45 ± 1	41 ± 3
HRT [day]	10 ± 1	10 ± 1
OLR [kgCOD/(m^3^ d)]	3.2 ± 1.7	3.4 ± 0.2
T [°C]	50 ± 2	48 ± 2
pH [-]	6.7 ± 0.1	7.1 ± 0.4

TS: total solids; VS: volatile solids; HRT: hydraulic retention time; OLR: organic loading rate; T: temperature.

## Data Availability

Not applicable.

## Supplementary Materials

The following are available online at https://www.mdpi.com/article/10.3390/membranes11120977/s1. Table S1: NUR values obtained from diverse carbon sources.

## Abbreviations

BSS	Biological sewage sludge
CAS	Conventional activated sludge
DBSS	Digested biological sewage sludge
HRT	Hydraulic retention time
NUR	Nitrate uptake rate
OLR	Organic loading rate
OUR	Oxygen uptake rate
ThAlMBR	Thermophilic alternate membrane biological reactor
TBSS	Thickened biological sewage sludge
TN	Total nitrogen
TS	Total solids
UF	Ultrafiltration
VS	Volatile solids
WW	Wastewater
WWTP	Wastewater treatment plant
