# Supplementary material for: How to Produce an Alternative Carbon Source for Denitrification by Treating and Drastically Reducing Biological Sewage Sludge
Source: Membranes (Basel). 2021 Dec 12;11(12):977. doi: 10.3390/membranes11120977 (PMC8708590; doi:10.3390/membranes11120977)
Supplement: Supplementary file 1 [file membranes-11-00977-s001.zip › membranes-1471220-supplementary.pdf]

membranes

MDPI

Article

How to Produce an Alternative Carbon Source for Denitrification by Treating and Drastically Reducing Biological Sewage Sludge

Maria Cristina Collivignarelli [1,2,*], Alessandro Abbà [3], Francesca Maria Caccamo [1], Marco Carnevale Miino [1], Angela Durante [4], Stefano Bellazzi [1], Marco Baldi [5] and Giorgio Bertanza [3]

1 Department of Civil Engineering and Architecture, University of Pavia, via Ferrata 3, 27100 Pavia, Italy; francescamaria.caccamo01@universitadipavia.it (F.M.C.); marco.carnevalemiino01@universitadipavia.it (M.C.M.); stefano.bellazzi01@universitadipavia.it (S.B.)
2 Interdepartmental Centre for Water Research, University of Pavia, Via Ferrata 3, 27100 Pavia, Italy
3 Department of Civil, Environmental, Architectural Engineering and Mathematics, University of Brescia, Via Branze 43, 25123 Brescia, Italy; alessandro.abba@unibs.it (A.A.); giorgio.bertanza@unibs.it (G.B.)
4 Freelance Chemist, Via Carducci 12, Casirate d'Adda, 24040 Bergamo, Italy; angela.durante@pec.chimici.it
5 Department of Chemistry, University of Pavia, Viale Taramelli 12, 27100 Pavia, Italy; marco.baldi@unipv.it
* Correspondence: mcristina.collivignarelli@unipv.it

Supplementary Materials

Table S1. NUR values obtained from diverse carbon sources.

Carbon Source	NUR [$mg_{N-NO3^-}/(g_{VSS}\ h)$]	References
Organic waste from soft drinks factory	31.8–48.1	[1]
Organic waste from sweet factory	30.4–41.6	[1]
Acetic acid	6.1 *	[2]
Industrial WW (effluents from brewery, distillery, and fish-pickling factory)	2.6–6.0	[3]
Methanol	3.2 *	[2]
Alcohol-based substance	3.0	[2]
Glycerine-based substance	2.4 *	[2]
ThAlMBR permeate	1.9 ± 0.3	[4]
Municipal sewage	0.6–1.0	[5]

* In the original study it is reported in [$mg_{N-NO3^-}/(g_{VSS}\ min)$]. NUR: nitrate uptake rate; WW: wastewater; ThAlMBR: thermophilic alternate membrane biological reactor.

References

1. Fernández-Nava, Y.; Marañón, E.; Soons, J.; Castrillón, L. Denitrification of high nitrate concentration wastewater using alternative carbon sources. *J. Hazard. Mater.* **2010**, *173*, 682–688.
2. Liwarska-Bizukojć, E.; Chojnacki, J.; Klink, M.; Olejnik, D. Effect of the type of the external carbon source on denitrification kinetics of wastewater. *Desalin. Water Treat.* **2018**, *101*, 143–150.
3. Swinarski, M.; Makinia, J.; Czerwionka, K.; Chrzanowska, M. Industrial wastewater as an external carbon source for optimization of nitrogen removal at the "Wschod" WWTP in Gdansk (Poland). *Water Sci. Technol.* **2009**, *59*, 57–64.
4. Collivignarelli, M.C.; Abbà, A.; Castagnola, F.; Bertanza, G. Minimization of municipal sewage sludge by means of a thermophilic membrane bioreactor with intermittent aeration. *J. Clean. Prod.* **2017**, *143*, 369–376.
5. Foladori, P.; Andreottola, G.; Ziglio, G. *Sludge Reduction Technologies in Wastewater Treatment Plants*; IWA Publishing: London, UK, 2010.
